# Anterior Pituitary Aplasia in an Infant with Ring Chromosome 18p Deletion

**DOI:** 10.1155/2016/2853178

**Published:** 2016-10-24

**Authors:** Edward J. Bellfield, Jacqueline Chan, Sarah Durrin, Valerie Lindgren, Zohra Shad, Claudia Boucher-Berry

**Affiliations:** ^1^Division of Pediatric Endocrinology, University of Illinois College of Medicine, Chicago, IL 60612, USA; ^2^University of Illinois College of Medicine, Chicago, IL 60612, USA; ^3^Department of Pathology, University of Illinois College of Medicine, Chicago, IL 60612, USA; ^4^Division of Genetics, University of Illinois College of Medicine, Chicago, IL 60612, USA

## Abstract

We present the first reported case of an infant with 18p deletion syndrome with anterior pituitary aplasia secondary to a ring chromosome. Endocrine workup soon after birth was reassuring; however, repeat testing months later confirmed central hypopituitarism. While MRI reading initially indicated no midline defects, subsequent review of the images confirmed anterior pituitary aplasia with ectopic posterior pituitary. This case demonstrates how deletion of genetic material, even if resulting in a chromosomal ring, still results in a severe syndromic phenotype. Furthermore, it demonstrates the necessity of close follow-up in the first year of life for children with 18p deletion syndrome and emphasizes the need to verify radiology impressions if there is any doubt as to the radiologic findings.

## 1. Introduction

18p deletion syndrome is caused by loss of all or parts of the short arm of chromosome 18 and is estimated to occur in approximately 1 in 50,000 live births [1]. The resultant syndrome has high phenotypic variability but is generally characterized by dysmorphic facies as well as congenital heart defects, isolated growth hormone (GH) deficiency, hypopituitarism, autoimmune conditions, and holoprosencephaly (HPE) [2]. While many of these cases are due to terminal deletions of the chromosome, other reported cases include unbalanced translocations, malsegregation of a balanced parental translocation, or a ring chromosome [1, 3, 4]. Here we present the first reported case of a patient with 18p deletion due to a ring chromosome with anterior pituitary aplasia.

## 2. Case Presentation

**Figure 1:**
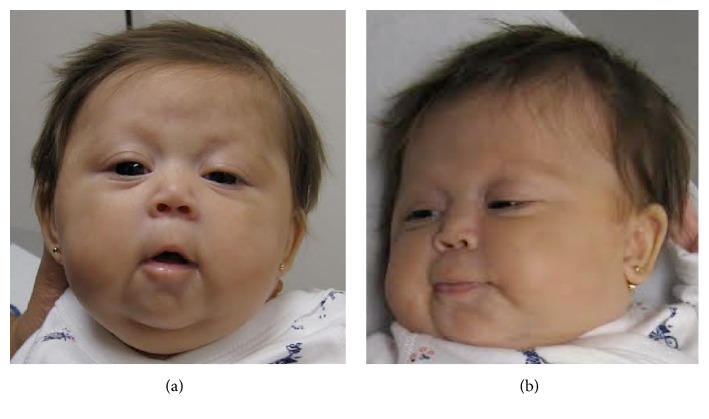
(a) Demonstration of round face, hypotelorism, and left-sided ptosis. (b) Demonstration of low set ear and flattened midface.

**Figure 2:**
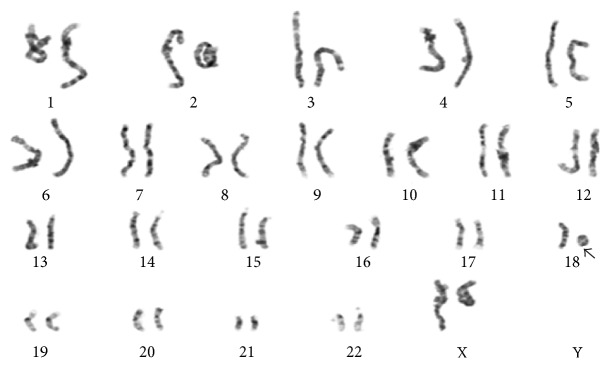
The patient's karyogram demonstrates a ring chromosome 18.

We present a female infant born at term to a 28-year-old G7P2 mother. The patient was prenatally diagnosed with a cystic hygroma and pleural effusions but the mother declined amniocentesis. At delivery, the patient was noted to have multiple dysmorphic features including round face, hypotelorism, unilateral ptosis, flattened midface, small nose, low set ears, and generalized hypotonia. Her features are shown at 3 months of age (Figures 1(a) and 1(b)). She also had mild coarctation of the aorta and persistent patent ductus arteriosus (PDA) despite indomethacin therapy. Her birth was complicated by respiratory distress requiring oxygen support, as well as unconjugated hyperbilirubinemia, which responded well to triple phototherapy. The presence of so many physical abnormalities leads to suspicion of a genetic syndrome. A chromosome analysis was then performed, which demonstrated a ring chromosome 18 (Figure 2).

**Figure 3:**
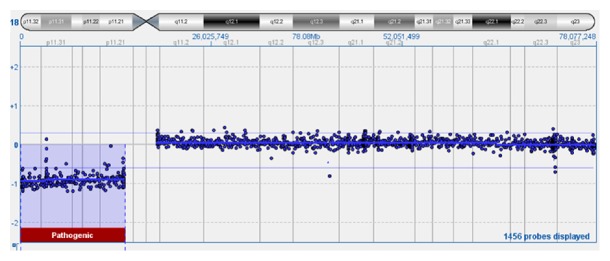
SNP based chromosome microarray confirmed a copy loss of ~13.93 Mb of 18p11.21p11.32, the entire short (p) arm of chromosome 18.

Single nucleotide polymorphism (SNP) chromosome microarray analysis confirmed copy loss of ~13.93 Mb of 18p11.21p11.32—the entire short (p) arm of chromosome 18 (Figure 3). No single copy sequences were deleted from the long arm. Therefore, this finding is equivalent to simple deletion of 18p with respect to gene content. A subsequent brain MRI was performed to evaluate for presence of associated HPE and was initially read as having no midline structural defect.

Endocrinopathies are known to be associated with 18p deletion syndrome, so initial lab workup was done at birth which demonstrated low insulin-like growth factor-1 (IGF-1) without hypoglycemia and normal thyroid function tests. The patient's feeding status continued to progressively decline leading to suboptimal weight gain. In part due to patient's worsening energy level, endocrine workup was repeated at 3 months of age.

These labs demonstrated undetectable IGF-1 and insulin-like growth factor-binding protein 3 (IGF-BP3), low free thyroxine (T4) with an inappropriately normal thyroid stimulating hormone (TSH), weak cortisol response post-1 hour adrenocorticotropic hormone (ACTH) stimulation test, and normal electrolytes without polyuria (Table 1). These findings were consistent with growth hormone deficiency, central thyroid deficiency, and secondary adrenal insufficiency, respectively—the hallmarks of anterior panhypopituitarism. She was started on hydrocortisone and later on somatropin and levothyroxine. It is highly likely that she will also exhibit gonadotropin deficiency, requiring hormonal supplementation to induce and maintain pubertal development.

**Figure 4:**
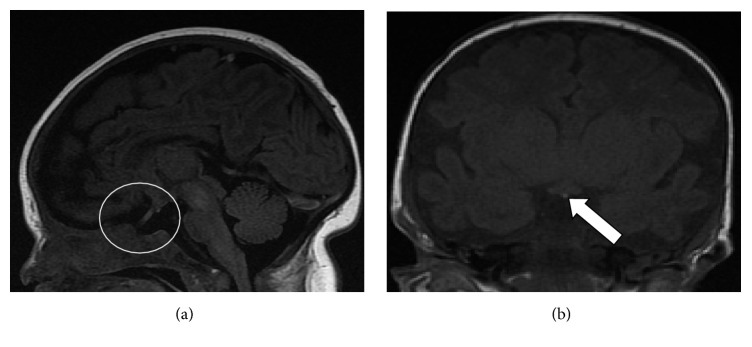
(a) MRI. Sagittal view demonstrates no evidence of the sella turcica and no pituitary soft tissue within the presumed area of the sella. (b) MRI. Coronal view demonstrates a superiorly displaced T1 bright spot consistent with an ectopic posterior pituitary.

A gastrostomy tube was placed to supplement oral feeds. Only after demonstrating appropriate weight gain did she undergo an uncomplicated PDA repair. Upon review of the initial imaging studies months later, it was concluded that she in fact did have an undeveloped sella turcica with no evidence of an anterior pituitary. The posterior pituitary bright spot was visible but displaced superiorly, which was expected due to a lack of symptoms of diabetes insipidus (Figures 4(a) and 4(b)).

## 3. Discussion

We present a patient with an 18p deletion syndrome secondary to ring chromosome, who exhibited phenotypic features similar to those described straightforward deletions of chromosome 18p. To our knowledge, there have been relatively few cases of isolated 18p deletion syndrome due to ring chromosome 18 described in the literature [3, 5, 6]. One significant aspect in which our patient differs from previously reported 18p deletion syndrome due to ring chromosome is the presence of anterior pituitary aplasia with posterior pituitary ectopy.

Since 18p deletion syndrome was first described by de Grouchy and colleagues in 1963 [7], a fairly comprehensive set of phenotypic effects due to breaks occurring in the short arm have been described. The most common features include neonatal complications (jaundice, respiratory distress, and feeding difficulties), hypotonia, and dystonia. Also frequently noted are facial dysmorphisms, ptosis, refractive errors, strabismus, and conductive hearing loss. HPE and its microforms have been observed in approximately 12% of these patients [8]. Interestingly, HPE has been linked to GH deficiency and there is evidence that isolated pituitary hypoplasia and pituitary stalk interruption syndrome are milder forms of HPE [6, 9]. Reports have varied on the developmental and behavioral manifestations, although recent reviews estimate an average IQ of 69, with patients ranging from mild impairment to normal functioning [2]. However regardless of IQ, many studies have noted that these individuals have difficulty with communication skills, activities of daily living, and management of social and occupational activities [2, 10].

Isolated hormone deficiencies and hypopituitarism have all been described as a feature of 18p deletion syndrome. Approximately 50% of breaks on chromosome 18 occur at the centromere, and while there are reports of direct parent-to-child transmission, approximately 70–85% of cases are due to de novo mutations [1, 2]. The remaining 50% of cases are due to an assortment of insults along the short arm of chromosome 18, adding to the phenotypic variability of this syndrome [3].

While GH deficiency has been previously described in both isolated 18p deletion syndrome and ring chromosome 18, the presentation is variable. Of the case reports describing GH deficiency associated with isolated 18p deletion syndrome, 2 of 3 patients had normal brain MRI [10, 11]. The third case report presented a patient with only partial GH deficiency and empty sella with rudimentary pituitary stalk [12]. Reported cases of GH deficiency in patients with ring 18 chromosome reveal inconsistent brain imaging results [5, 11, 13]. Additionally, a female with a ring 18 chromosome, GH deficiency, hypothyroidism, and ectopic posterior pituitary (but normal cortisol) has been described [14].

The most recent review of 18p deletion syndrome [2] cited that approximately 23% of patients had isolated GH deficiency, while 13% had hypopituitarism or panhypopituitarism. In the authors' own cohort of 54 patients, 6 had pituitary abnormalities, including hypoplastic pituitary or pituitary stalk, absent posterior pituitary, or complete pituitary absence. Additionally, 5 of these 54 had hypothyroidism without structural abnormalities. Another recent review of patients with ring chromosome 18 described similar findings: 93% of patients had isolated GH deficiency, 41% had hypothyroidism, and 13% had a spectrum of HPE [15].

Current research in 18p deletion syndrome is aimed at establishing gene-specific phenotypic correlations and determining the penetrance of these phenotypes in order to provide genotype-specific anticipatory guidance for these patients and their families [2]. While there is a clear association between 18p deletion syndrome and endocrinopathies and structural pituitary abnormalities, the specific genes responsible for these phenotypes have yet to be identified. Our hope is that case reports such as this in addition to further molecular characterization of 18p deletion genotypes will help to further elucidate the mechanism behind this condition and allow for improved care and treatment.

## 4. Conclusion

This is the first reported case of an infant with an 18p deletion syndrome secondary to a ring chromosome with anterior pituitary aplasia and ectopic posterior pituitary. Although endocrinology labs soon after birth were reassuring, repeat testing months later identified central endocrinopathy and review of the imaging confirmed the diagnosis. This case demonstrates the necessity of close follow-up in the first year of life for children with 18p deletion syndrome but also emphasizes the need to verify radiology impressions if there is any doubt as to the radiologic findings.

## Figures and Tables

**Table 1:** Endocrinology workup obtained at day of life 22, compared with that obtained at day of life 100 (3 months of age).

Lab test	DOL 22	DOL 100	Reference values
IGF-1	16	<1	56–124 ng/mL
IGF-BP3	—	<500	1039–3169 ng/mL
Cortisol	3.2 (random)	8 (post-ACTH)	≥18 *μ*g/dL (post-ACTH)
TSH	6	2.2	0.35–10 *μ*IU/mL
FT4	0.7	0.5	0.6–1.7 ng/mL
